# Supplementary material for: The Pattern of Progression to First-Line Treatment with Dabrafenib and Trametinib in Patients with Unresectable or Metastatic, BRAF-Mutated, Cutaneous Melanoma: Results of the Observational T-WIN Study
Source: Cancers (Basel). 2023 Mar 26;15(7):1980. doi: 10.3390/cancers15071980 (PMC10093702; doi:10.3390/cancers15071980)
Supplement: Supplementary file 1 [file cancers-15-01980-s001.zip › Table.pdf]

**Table S1 Clinical benefit according to metastatic site at baseline.** Data were reported as median and 95% confidence intervals.

| **Cohort A** | | | **Cohort B** | | |
|---|---|---|---|---|---|
| | **OS** | **PFS** | | **OS** | **PFS** |
| Liver (n=26) | 20.6 months (9.9-NE) | 8.9 (6.6-12.5) | Liver (n=46) | 6.8 (5.6-8.2) | 5.7 (4.2-6.6) |
| Brain (n=14) | NE (8.2-NE) | 12.5 (6.6-NE) | Brain (n=37) | 8.3 (6.6-11.7) | 6.9 (5.2-9.4) |
| Skin (n=16) | NE | NE | Skin (n=16) | 10.3 (5.7-14.0) | 7.0 (4.0-9.9) |

**Table 2S Second-line treatment**

| | **Cohort A (n=46)** | **Cohort B (n=32)** |
|---|---|---|
| Pembrolizumab | 9 | 5 |
| Nivolumab | 19 | 23 |
| Ipilimumab | 1 | 2 |
| Encorafenib | 2 | 1 |
| Binimetinib | 2 | 1 |
| Not specified | 13 | 0 |
