# Supplementary figures and images for: The Pattern of Progression to First-Line Treatment with Dabrafenib and Trametinib in Patients with Unresectable or Metastatic, BRAF-Mutated, Cutaneous Melanoma: Results of the Observational T-WIN Study
Source: Cancers (Basel). 2023 Mar 26;15(7):1980. doi: 10.3390/cancers15071980 (PMC10093702; doi:10.3390/cancers15071980)

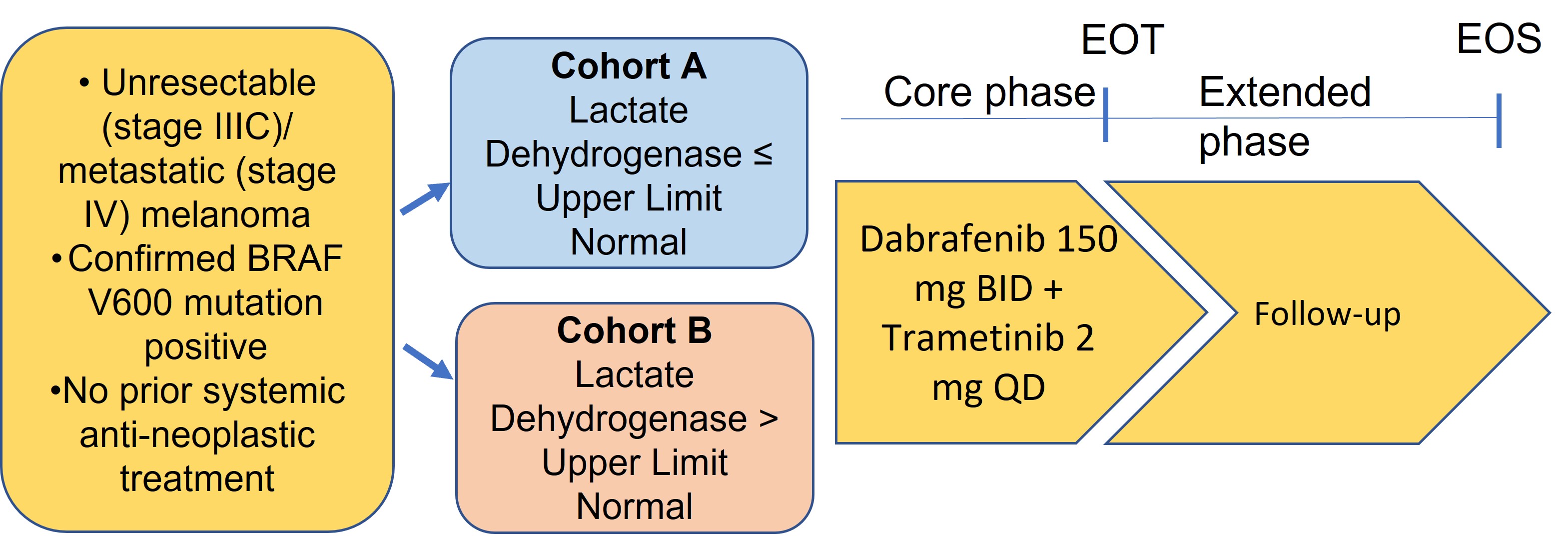

Supplement: Supplementary file 1 [file cancers-15-01980-s001.zip › Figure 1S.jpg]

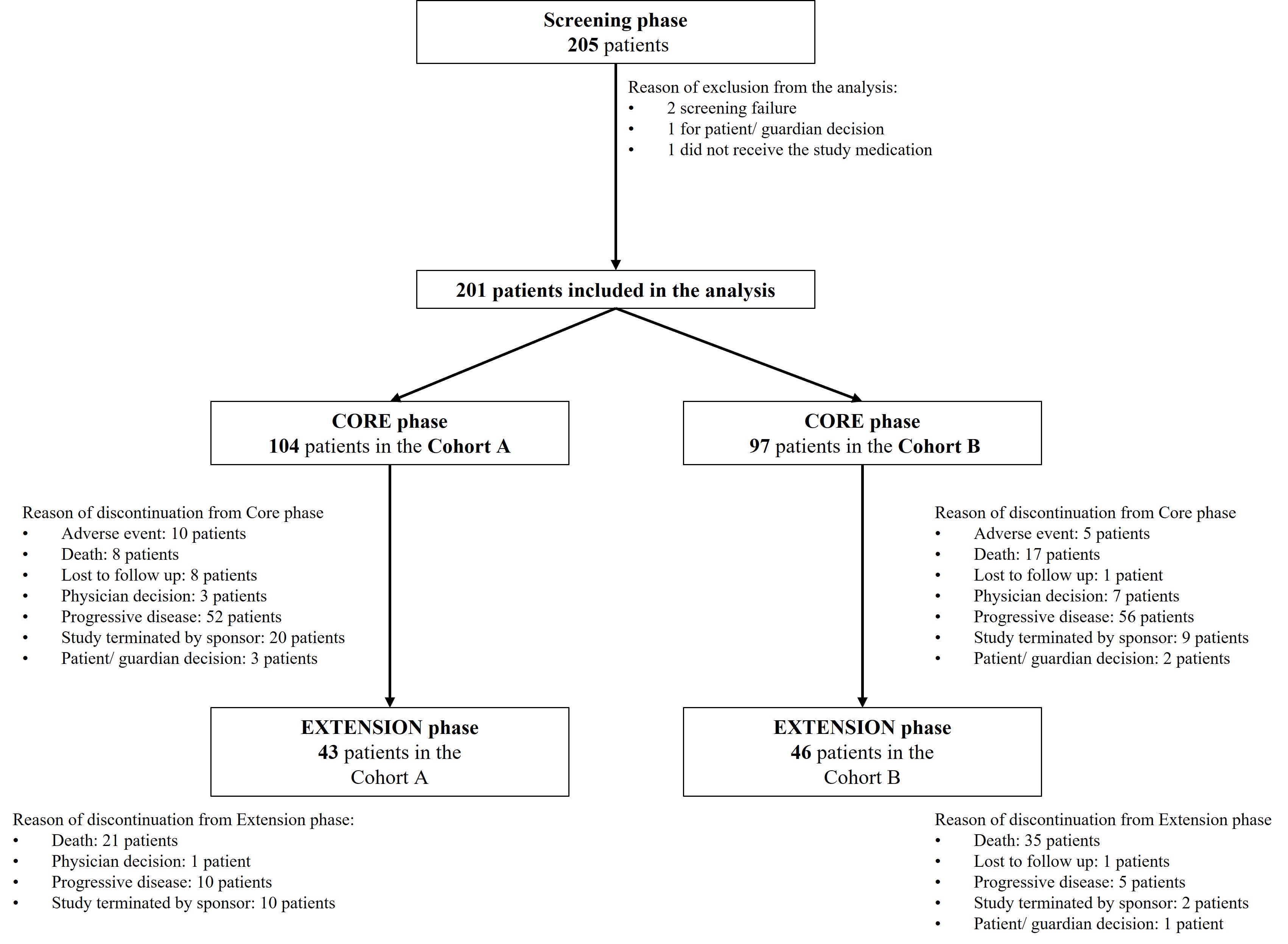

Supplement: Supplementary file 1 [file cancers-15-01980-s001.zip › FIgure 2S.jpg]
